# Effects of perceived English teacher support on student engagement among Chinese EFL undergraduates: L2 motivational self system as the mediator

**DOI:** 10.3389/fpsyg.2025.1607414

**Published:** 2025-05-19

**Authors:** Wenxing Zhang, Jiayue Hu

**Affiliations:** School of Foreign Languages, Jiangxi Science and Technology Normal University, Nanchang, Jiangxi, China

**Keywords:** perceived teacher support, student engagement, learning motivation, the mediating effect, EFL learning

## Abstract

**Introduction:**

Student engagement is a vital evaluation index in the learning process of English learners and serves as a key indicator of the effectiveness and quality of college English teaching.

**Methods:**

Based on the perspective of the second language motivational self system theory, this study used a structural equation model to explore the general profile of 2,633 Chinese undergraduates of English as a foreign language (EFL) perceived teacher support, second language (L2) motivation, and student engagement in English classrooms, and the interrelationships among the three variables.

**Results:**

The results showed that teacher support and student engagement were at high levels, and L2 motivation was at a medium-high level in English learning. Perceived teacher support significantly correlated with L2 motivation, and their profound influence on student engagement. The indirect effect results showed that the L2 motivational self system mediated the effect of perceived teacher support on student engagement, and L2 learning experience exerted a greater mediating effect compared to the ideal L2 self and ought-to L2 self.

**Discussion:**

These findings deepen the understanding of the interaction mechanism between these variables and provide helpful references to improve student engagement and learning effectiveness.

## 1 Introduction

In the era of globalization and informatization, mastering English has become an important driving force for personal development and social progress. However, the process of English learning is full of challenges, and how to motivate learners and maintaining their engagement has become an important issue in education. Motivation is crucial to English learning as a driving factor and key to success (Li and Zhang, 2021). Inadequate motivation can lead to failure to complete learning tasks, which in turn affects the achievement of long-term learning goals (Dörnyei, 2020). Learning English as a foreign language in China presents a unique context. On the one hand, China’s examination-oriented education system significantly shapes students’ L2 motivation and engagement (Hu et al., 2025), fostering an instrumental motivation primarily focusing on achieving high grades in exams, which may undermine intrinsic motivation and consequently diminish sustained student engagement. On the other hand, English proficiency has transcended the realm of mere language skills, and is a key competency for students to engage in international exchanges and broaden their global horizons. Therefore, it is of great value to explore how to stimulate students’ L2 motivation and student engagement in the Chinese educational context.

In recent years, the L2 motivational self system theory has provided a new perspective for understanding motivation in English learning, emphasizing the driving roles of the ideal L2 self, the ought-to L2 self, and the L2 learning experience on learning behaviors, which influences student engagement and learning effectiveness (Papi, 2010). Meanwhile, teacher support, as one of the important factors in the school context, is considered to have a significant impact on motivating students and enhancing student engagement (Affuso et al., 2023; Luan et al., 2023). On the other hand, student engagement, as a vital indicator of learners’ actual participation and effort in learning activities, is essential in ensuring learning effectiveness. Student engagement is a dynamic process (Sulis, 2024) and is influenced by a variety of elements, among which both teacher support and L2 self-motivation play an prominent function (Papi, 2010; Strati et al., 2017). However, the studies on the relationship between perceived teacher support, L2 motivational self system, and student engagement are relatively limited, particularly among Chinese non-English-major undergraduates, and this issue has not yet been fully explored.

Therefore, this very study aims to examine the interaction between perceived English teacher support, L2 motivational self system, and student engagement among Chinese EFL undergraduates. Constructing a mediation model to analyze how teacher support promotes student engagement through the L2 motivational self system, to provide a theoretical foundation and practical insights for improving student engagement and learning effectiveness.

### 1.1 Perceived English teacher support

Teachers are often recognized as a vital factor in L2 teaching and learning contexts (Liu and Li, 2023), exerting a significant influence on students’ learning, including academic achievement (Brandisauskiene et al., 2021), academic emotions (Jiang and Dewaele, 2019; Sadoughi and Hejazi, 2021), learning motivation (Brandmiller et al., 2024), and student engagement (An et al., 2022). Specifically, English teachers cultivate affirmative teacher-student interactions (Robinson, 2022) and provide students with enough support to build a safe and encouraging environment (Furrer and Skinner, 2003) to increase student engagement and achievement.

Teacher support is an important component of the social support system and can be viewed from both a broad and narrow perspective. The broad perspective depends on the framework of social support (Tardy, 1985), which defines teacher support as a set of supports, such as advice, trust, and resources, that teachers provide to students. The narrow perspective sees teacher support as limited to help, trust, and interest in the classroom contest (Aldridge et al., 1999). Ryan and Patrick (2001) defined teacher support as the extent to which students trust teachers to value them and build interpersonal relationships with them. Synthesizing the existing studies and the characteristics of foreign language teaching in Chinese universities, this study classified English teacher support into three categories: emotional support, competence support, and autonomy support. Teacher emotional support defines teachers’ concern and respect for students’ learning and daily life (Patrick et al., 2007). Teacher competence support is also regarded as structural support, which refers to clear and specific expectations, instruction, guidance, support, and constructive feedback from teachers to students (Jang et al., 2010). Teacher autonomy support is expressed as giving students freedom in learning activities, establishing a connection between learning activities and students’ interests, and refraining from exerting external control and pressure (Skinner and Belmont, 1993).

From the perspective of ecological systems theory, student engagement is strongly linked to the learning context (Fredricks et al., 2004). When the learning context satisfies the basic psychological needs of individual learners, learners show higher engagement and achieve better learning outcomes (Ryan and Deci, 2009). Teacher support is of crucial importance in this process, and the study conducted by Jang et al. (2010) applying observational evaluation methods indicated that both teacher autonomy and structural support predicted student engagement. Zhou et al. (2023) further stated that effective teacher support serves as a vital indicator of teacher-student interrelationships and promotes student engagement in the classroom. When the learning context meets the psychological needs of individual learners, it is effective in stimulating motivation (Schweder and Raufelder, 2024). Specifically, teacher emotional support can increase English learning motivation (Ruzek et al., 2016) as well as student self-efficacy (Yang et al., 2022). In addition, existing research has revealed a strong association between teacher support and motivation (Chiu et al., 2024). However, existing studies have revealed a strong connection between teacher support and learning motivation (Brandmiller et al., 2024; Chiu et al., 2024). Besides, prior studies have concentrated on self-determination theory (Zhang and Liu, 2022), and less attention has been paid to L2 motivational self system theory. Given English globalization, the theory emphasizes the learner autonomy, the interaction between individuals and collectives, and the dynamic changes in psychological structure (Al-Hoorie, 2018). In China’s socio-cultural context, students are often driven by achievement-oriented mindsets, such as the desire to be a good student or get good grades (Hu et al., 2025). The ideal L2 self and the ought-to L2 self of L2 motivational self system theory both belong to the self-guided category (Dörnyei and Ushioda, 2009), and are the desired height of students, which can effectively explain their achievement-driven motivational characteristics. As an important theory of foreign language learning motivation, the L2 motivational self system theory is in line with the characteristics of the national language context in China, where English is learned as a foreign language (Xu and Wang, 2022). Therefore, exploring the function of teacher support in this theoretical framework will contribute to deepening the understanding of L2 motivational self system theory and providing more sound support strategies for teaching. Therefore, based on the existing literature, the first research hypothesis is proposed.

*H1a:* Perceived teacher support positively predicts ideal L2 self.

*H1b:* Perceived teacher support positively predicts L2 learning experience.

*H1c:* Perceived teacher support positively predicts ought-to L2 self.

### 1.2 L2 motivational self system

Learning motivation is considered to play a critical role in influencing the commitment to foreign language behavioral engagement (Dörnyei and Ushioda, 2009; Zhu et al., 2024). Early studies generally adopted the “integrative motivation” and “instrumental motivation” proposed by Gardner and Lambert (1959). However, with the development of the times and the depth of research, more and more scholars began to realize the inadequacy of Gardner’s study, and Yashima (2002) proposed the “international posture” instead of the traditional “integrative motivation.” Consequently, founded on the inheritance of traditional motivation theories against the global context, Dörnyei (2005) pointed out the insufficiency of the concept of “integrative” and “instrumental” motivation, and proceeded to develop the traditional theory of motivation by incorporating the conception of the “self” (Higgins, 1987; Markus and Nurius, 1986) in psychology, the introduction of the characteristics of “time,” and the “learning environment.”

Therefore, the L2 motivational self system theory was proposed, which links the self theory and identity theory with a new conceptual framework, placing the “self” at the center of motivation and behavior research. It includes: the ideal L2 self as the core concept, which refers to the ideal self-image that learners would like to have to become proficient in L2, to motivate them to strive to close the gap between their ideal learning achievement and current learning level, and it includes the traditional classification of integrative motivation and internalized instrumental motivation (Fathi and Hejazi, 2024). Ought-to L2 self is a characteristic that learners are often required to meet the expectations of others (e.g., family members, friends, teachers) or to avoid the negative results caused by not learning a foreign language well, which more corresponds to the external instrumental motivation (Boo et al., 2015). L2 learning experience is a specific contextual factor that encourages students to participate in language learning (Rahimi and Sevilla-Pavón, 2025). Dörnyei (2009) argued that for some language learners, initial learning motivation is mainly derived from successful learning experiences in the initial stages of L2 learning and may be influenced by specific factors in the learning context, such as teachers, peers, and learning materials. Numerous studies have proved that this theory has high reliability and validity (Taguchi et al., 2009; Xu and Wang, 2022).

In recent years, empirical studies have explored that the L2 motivational self system has a strong predictive effect on students’ foreign language learning behaviors, and can motivate them to learn English more diligently and with more engagement (Papi, 2010). Among them, the ideal L2 self and positive L2 learning experiences are the key factors motivating students to be more engaged in foreign language learning (Taguchi et al., 2009) and improve their academic performance (Li and Zhang, 2021). Dörnyei and Chan (2013) further pointed out that the stronger the motivation of the ideal L2 self, the more positive the students’ motivational behaviors, thus contributing to higher English achievement. However, regarding the role of the ought-to L2 self, empirical findings are inconsistent, with three scenarios of facilitation (Al-Hoorie, 2018; Li et al., 2025), hindrance (Li and Zhang, 2021), and no significant effect (Tahmouresi and Papi, 2021). Therefore, the relationship between the ought-to L2 self and student engagement remains inconclusive. In China’s collectivism-oriented educational context, individuals are particularly susceptible to external expectations and norms, and this cultural context significantly affects students’ motivation (Hu et al., 2025). Correspondingly, a profound understanding of what ought-to be L2 self input to learning in the Chinese EFL context is very important. Additionally, the L2 motivational self system has been extensively explored in second language acquisition, yet focusing on the interaction between the multi-dimensionality of the L2 motivational self system and student engagement in the Chinese EFL context carries great theoretical and practical significance. Then, this study introduces the second hypothesis.

*H2a:* The ideal L2 self positively predicts student engagement.

*H2b:* The L2 learning experience positively predicts student engagement.

*H2c:* The ought-to self positively predicts student engagement.

### 1.3 Student engagement

Student engagement is an important indicator to measure the degree of learner participation in learning tasks (Reeve, 2012), as well as an important measure of learning quality, which positively predicts academic performance and reflects the behavioral, cognitive, and emotional effort learners put into learning. In the context of foreign language learning, the role of student engagement is particularly important because the automation of language skills requires the persistent and full engagement of learners (Hiver et al., 2020). As a multidimensional concept, the classical model of student engagement, which is based on the three-dimensional structure of behavior, cognition, and emotion (Fredricks et al., 2004), has been widely utilized for second language acquisition research (Xu J. et al., 2023), providing theoretical support for understanding the investment in foreign language learning. Among them, behavioral engagement refers to the degree of effort during the learning process and the positive degree of participation in learning events (Philp and Duchesne, 2016). Cognitive engagement emphasizes the degree of engagement in how they make use of various self-regulation and learning strategies during the learning process (Xu J. et al., 2023). Emotional engagement is defined as the emotional experience and affective attitudes that students perceive in the process of learning activities, including positive and negative emotions (Sulis, 2024).

However, student engagement differs across individuals, disciplines, and learning contexts, and the level and quality of engagement can be affected by a variety of factors. At the individual learner level, it includes students’ background information (Angelovska et al., 2021), motivation (Yang and Du, 2023), and academic emotions (Dewaele and Li, 2021). In the external environment aspect, the teacher-student relationship (Engels et al., 2021), teacher support (Sadoughi and Hejazi, 2021), and school context (Cayubit, 2022) are also influential factors. Akram and Li (2024) examined that teacher-student relationship and motivation have a positive consequence on student engagement, and intrinsic motivation has the greatest mediating role between teacher-student relationship and student engagement. In general, student engagement can be influenced by personal and environmental factors; therefore, using L2 self-motivation as a mediator can comprehensively examine the specific mechanisms by which teacher support affects student engagement, which is important for improving the level of student engagement and the quality of foreign language teaching. Then, the third hypothesis is formulated.

*H3:* Perceived teacher support positively predicts student engagement.

### 1.4 Correlates of perceived teacher support, L2 motivational self system, and student engagement

Teachers’ supportive behavior and the students’ L2 motivation are both crucial factors in the English teaching ecological systems. Teacher support significantly influences the ideal L2 self, ought-to L2 self, and L2 learning experience (Elsayed et al., 2024; Zhang and Liu, 2022), and can directly predict student engagement. Whereas the ideal L2 self, ought-to L2 self, and L2 learning experience independently predict student engagement (Dörnyei and Chan, 2013; Ghasemi, 2023). This indicated that the L2 motivational self system may mediate the relationship between teacher support and student engagement. Specifically, by providing various forms of support, teachers can help students develop positive L2 motivation for L2 learning, thus promoting student engagement (Ghasemi, 2023). Given the relationship between the three constructs, this study proposes the fourth hypothesis.

*H4.* Ideal L2 self, ought-to L2 self, and L2 learning experience mediate the relationship between perceived teacher support and student engagement.

**Figure 1:**
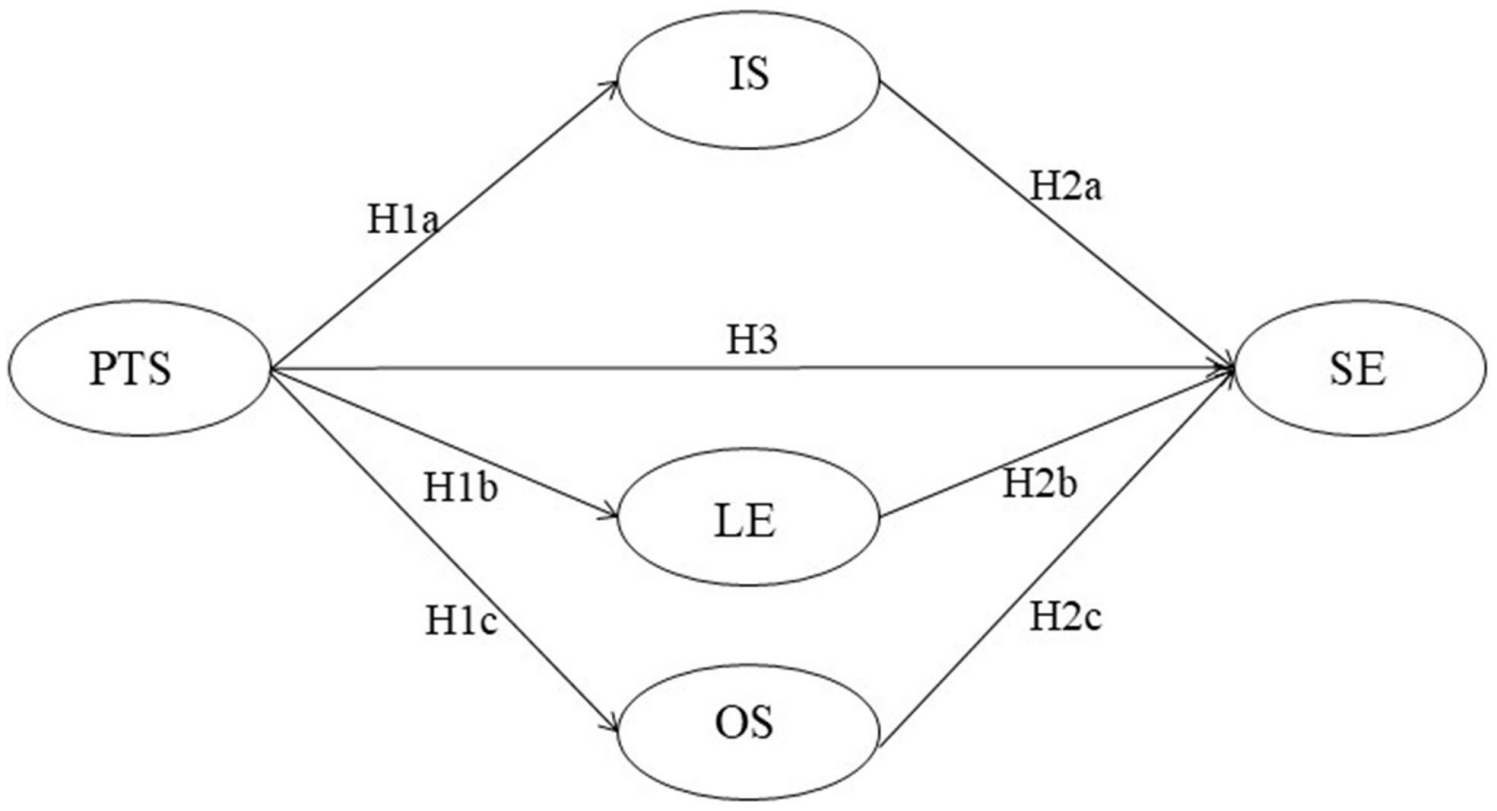
Hypothesized model. PTS, perceived teacher support; SE, student engagement; IS, ideal L2 self; OS, ought-to L2 self; LE, L2 learning experience.

To sum up, although existing studies have confirmed the correlation between teacher support, L2 motivation, and student engagement, the underlying mechanisms of the three variables remain under-explored, especially the mediating effect of the L2 motivational self system. Given the importance of student engagement, teacher support, and L2 motivation in English learning, the present study is based on the L2 motivational self system theory, focusing on Chinese EFL undergraduates, and aims to explore the latent interactions among these variables. Additionally, special attention is paid to the mediating role of the L2 motivational self system (Figure 1). Therefore, this study aims to address the following three research questions:

1. What are the Chinese EFL undergraduates’ levels of perceived English teacher support, L2 motivational self system, and engagement in English classrooms?
2. What are the interactions between Chinese EFL undergraduates’ perceived English teacher support, L2 motivational self system, and engagement in English classrooms?
3. To what extent do Chinese EFL undergraduates perceive English teacher support contributing to student engagement, taking into account the mediating role of the L2 motivational self system?

## 2 Research method

This study utilized a quantitative research method within the Chinese context, employing a questionnaire survey. In this approach, numerical data about the research participants were gathered through questionnaires, followed by a quantitative analysis of the collected data. It also constructed a complex mediation model describing the direct and indirect effects of an independent variable (perceived teacher support), three mediation variables (ideal L2 self, ought-to L2 self, and L2 learning experience), and one dependent variable (student engagement).

### 2.1 Research participants

The convenience sampling approach was used to recruit non-English major undergraduates from various colleges in Jiangxi Province in southeastern China as the research participants because it was more affordable, and participants were readily available (Taherdoost, 2016). In all, 3,236 students anonymously took part in the questionnaire survey.

For the formal survey, the researchers contacted the teachers of relevant universities and, with the consent of the teachers, distributed and collected the questionnaires uniformly through the Wenjuanxing.^1^ In alignment with rigorous ethical practices, anonymity was stringently maintained in all responses, and all responses were handled under strict confidentiality protocols. After the data were obtained, they data were cleaned. Specifically, participants whose response time was less than 2 s per item were excluded, as a response time of 2 s per item is considered a benchmark for ensuring attention and response quality (Huang et al., 2012). In addition, participants who provided a range of identical answers equal to the number of items were also removed (Curran, 2016). A total of 2,633 questionnaires were retrieved, and the recovery efficiency rate is 81.40%. The participants of the study included Chinese EFL undergraduates from Year 1 to Year 5 were 1930 (73.3%), 663 (25.2%), 29(1.1%), 8 (0.3%), and 3 (0.1%), respectively. Because college English is a compulsory course for these undergraduates during their four or five-year undergraduate study, and is usually offered in the first two years of university study. Due to academic pressure or future developments, students at this stage have more time to learn English than juniors or seniors. Therefore, they are more suitable as participants for this study. Among them, 740 male students accounted for 28.1%, and 1893 female students accounted for 71.9%, involving most of the majors of arts, science, engineering, and other disciplines. The participants’ ages ranged from 18 to 22 years old, and they had more than 9 years of formal English learning experiences. During the questionnaire survey, the students were informed of the principle of voluntary participation. Informed consent was acquired from the students, and they actively participated in the questionnaire. At the same time, to prevent comprehension bias, all the items in the questionnaire were presented in Chinese. The researchers strictly adhered to the ethical guidelines of the authors’ university throughout the entire research process.

### 2.2 Measures

The questionnaire in this study consisted of two parts: the first part focuses on students’ personal information, including gender, grade, major, and informed consent; the second part investigates the perceived English teacher support scale, L2 motivational self system scale, and student engagement scale. All questionnaires were presented on a five-point Likert scale with answers ranging from 1 (“completely disagree”) to 5 (“completely agree”).

#### 2.2.1 Perceived English teacher support scale

The Perceived English teacher support scale consists of three sub-scales: competence, emotional, and autonomy support. The competence support sub-scale was adapted from the scale by Chi (2017), the emotional support sub-scale was developed from the scale by Patrick et al. (2007), and the autonomy support sub-scale was derived from the scale by Chai and Gong (2013). These scales have been rigorously validated for reliability and validity, and have been localized and adapted according to the learning characteristics of Chinese EFL undergraduates, with good reliability and validity (An et al., 2022; Xu X. et al., 2023). The scales consisted of 10 items, including four for competence support (for example, “The English teacher provides constructive feedback on my learning performance.”), three for emotional support (for example, “The English teacher knows and cares about me.”), and three items for autonomy support (for example, “The English teacher gives us enough time for self-study or independent thinking.”).

#### 2.2.2 Student engagement scale

The student engagement scale used in this study was based on the College English Curriculum Student Engagement Questionnaire (Reeve and Tseng, 2011; Xu J. et al., 2023). Both studies involved Chinese university students as participants, who exhibited notable similarities in student engagement characteristics. The questionnaire, which was revised specifically for Chinese EFL undergraduates, was reported to possess good reliability and validity. The questionnaire segregates student engagement into three distinct dimensions: behavioral engagement (three items, for example, “I actively interact with my teachers and classmates in the English class.”), emotional engagement (three items, for example, “I feel very happy when I learn new knowledge in the English class.”), and cognitive engagement (four items, for example, “I take the initiative to find ways to solve the difficult problems in English learning.”).

#### 2.2.3 L2 motivational self system scale

The scale used was adapted from the scale (Papi, 2010; Taguchi et al., 2009). Some items were deleted and modified to better align with the actual situation of the Chinese students’ foreign language learning. This instrument has been proven to be reliable and valid among Chinese students (Lee and Lee, 2021). There are 12 items in the scale, including three dimensions: 4 for ideal L2 self (for example, “I can imagine myself writing English e-mails/letters fluently.”), 4 for ought-to L2 self (for example, “I consider learning English important because the people l respect think that I should do it.”), and 4 for L2 learning experience (for example, “I find learning English really interesting.”).

### 2.3 Research procedures

The survey was divided into two stages: pilot study and formal study. To test the structural validity and reliability of these scales, 487 Chinese EFL undergraduates from a college in Jiangxi, China, participated in the questionnaire survey. The results indicated that the standardized Cronbach’s alpha coefficients for the three scales were 0.941, 0.927, and 0.905. The KMO values for the validity tests were 0.944, 0.935, and 0.901, and Barlett’s spherical tests were all significant (*p* < 0.001), suggesting that the data were suitable for both exploratory and confirmatory factor analysis. Confirmatory factor analysis was utilized for these scales and entry in the L2 motivational self system scale should have a low factor (I consider learning English important because it will help me find a good job in the future.) loading (0.48) less than 0.50 (Hair et al., 2012) for the ought-to L2 self dimension, so it was deleted. The student engagement scale was subjected to confirmatory factor analysis as well. In the emotional engagement dimension, there were large residual correlations between EE4 and EE1 (88.176), EE4 and EE2 (16.525), and EE4 and EE3 (10.666) indicating that the EE4 item was similar to the other items, so the EE4 item was deleted, and the model fit was better after the deletion (Lance et al., 2010). After the deletion, which consisted of four items for ought-to L2 self and three items for emotional engagement.

In the formal study, the Cronbach’s alpha for the three scales was 0.963, 0.936, and 0.963. Confirmatory factor analyses further demonstrated that the fit indices of the three scales [CFI = 0.985 (> 0.90), TLI = 0.978 (> 0.90), GFI = 0.968 (> 0.90), SRMR = 0.010 (< 0.080); CFI = 0.970, TLI = 0.957, GFI = 0.951, and SRMR = 0.020; CFI = 0.958, TLI = 0.946, GFI = 0.939, SRMR = 0.041], indicated that the structural model fitted the data well (Marsh et al., 1988).

Table 1 showed the composite reliability (CR) and average variance extracted (AVE) values of the measurement model. The CR value greater than 0.70 and AVE value greater than 0.50 (Hair et al., 2019) indicate that the scale had good convergent and discriminant validity.

**Table 1:** The convergent and discriminant validity test.

Variables	Items	Std.loading	AVE	CR
ES	ES1	0.73	0.695	0.872
	ES2	0.864		
	ES2	0.898		
AS	AS1	0.873	0.769	0.909
	AS2	0.878		
	AS2	0.879		
CS	CS1	0.810	0.736	0.918
	CS2	0.883		
	CS3	0.875		
	CS4	0.862		
EE	EE1	0.752	0.589	0.811
	EE2	0.745		
	EE3	0.804		
BE	BE1	0.82	0.674	0.846
	BE2	0.767		
	BE3	0.824		
CE	CE1	0.799	0.706	0.906
	CE2	0.871		
	CE3	0.870		
	CE4	0.819		
IS	IS1	0.874	0.670	0.890
	IS2	0.902		
	IS3	0.746		
	IS4	0.740		
LE	LE1	0.765	0.737	0.918
	LE2	0.902		
	LE3	0.923		
	LE4	0.835		
OS	OS1	0.875	0.585	0.849
	OS2	0.679		
	OS3	0.798		
	OS4	0.774		

ES, Emotional support; AS, Autonomy support; CS, Competence support; BE, Behavioral engagement; EE, Emotional engagement; CE, Cognitive engagement; IS, ideal L2 self; OS, ought-to L2 self; LE, L2 learning experience; CR, Composite reliability; AVE, Average variance extracted.

### 2.4 Data collection and analysis

First, we conducted confirmatory factor analysis to assess whether the research results were affected by common method bias. Second, a normal distribution test, descriptive statistics, and Pearson correlation analysis were conducted using SPSS 26.0. Third, structural equation modeling using Amos 26.0 was used to investigate the multiple mediating effects of the L2 motivational self system.

## 3 Results

### 3.1 Common method bias test

The data were collected through self-reports by the participants, common methodological biases may have affected the results. To examine the common method bias, the study employed confirmatory factor analysis to conduct a one-factor model, which showed poor fit of the one-factor model and unsatisfactory model fitting results (X^2^/df = 50.083, CFI = 0.672, TLI = 0.649, GFI = 0.463, RMSEA = 0.137, SRMR = 0.081). The results of this test showed that common method bias is not serious. Then, the unmeasured latent method construct variance comparison (ULMC) approach was used to test for common method bias (Podsakoff et al., 2003). The study established a validated factor analytic model and a comparative model incorporating the common method factors, then compared the key fit indices of the two models (△RMSEA = 0.009, △RMR = 0.016, △TLI = 0.017, △CFI = 0.017, △GFI = 0.011). The results indicated that the model was not significantly improved after adding the common method factor, so this study has no significant common method bias.

### 3.2 Descriptive statistics

Table 2 presented that the skewness coefficients of all dimensions of the three variables are between plus and minus 3, and the kurtosis coefficients are between plus and minus 8, indicating that the variables are basically acceptable as normally distributed (Field, 2024). To answer the first research question, descriptive statistical analyses showed that Chinese EFL undergraduates perceived English teacher support (*M* = 4. 201, SD = 0. 625) was at a high level, with autonomy (*M* = 4. 321, SD = 0. 648) and competence support (*M* = 4. 252, SD = 0. 660) at a high level and emotional support (*M* = 4. 048, SD = 0. 706) at a slightly lower level. Among the three dimensions of student engagement (*M* = 3.987, SD = 0. 643), the average score of emotional engagement (*M* = 4. 051, SD = 0. 695) is the highest, behavioral engagement (*M* = 3.963, SD = 0. 706) is the second highest, and cognitive engagement (*M* = 3.945, SD = 0. 695) is the lowest. Meanwhile, learning motivation (*M* = 3.738, SD = 0. 689) for second language learning is at a moderately high level, and the average scores of L2 learning experience (*M* = 3.888, SD = 0. 764) and ideal L2 self (*M* = 3.779, SD = 0. 835)are higher than the ought-to L2 self (*M* = 3.547, SD = 0. 846).

**Table 2:** Descriptive statistics (*N* = 2,633).

Variable	Mean	SD	Variance	Skewness	kurtosis
Total teacher support	4.201	0.625	0.391	−1.279	4.027
Emotional support	4.048	0.706	0.498	−0.932	2.213
Autonomy support	4.321	0.648	0.420	−1.503	4.815
Competence support	4.252	0.660	0.436	−1.273	3.699
Overall engagement	3.987	0.643	0.413	−0.807	2.172
Behavioral engagement	3.963	0.706	0.498	−0.763	1.438
Emotional engagement	4.051	0.695	0.483	−0.931	2.203
Cognitional engagement	3.945	0.695	0.483	−0.732	1.646
L2 motivational self system	3.738	0.689	0.475	−0.459	0.890
Ideal L2 self	3.779	0.835	0.697	−0.710	0.621
Ought-to L2 self	3.547	0.846	0.716	−0.376	0.022
L2 learning experience	3.888	0.764	0.584	−0.730	0.961

### 3.3 Correlation analyses

As shown in Table 3, the Pearson correlation analysis addressed the second question. The results revealed that the dimensions of perceived English teacher support and student engagement were positively correlated (*r* = 0.531–0.708, *p* < 0.01). The L2 motivational self system was positively correlated with student engagement (*r* = 0.455–0.772, *p* < 0.01). The dimensions of perceived English teacher support showed a significant positive correlation with the dimensions of the L2 motivational self system (*r* = 0.287–0.580, *p* < 0.01).

**Table 3:** Correlation analysis (*N* = 2,633).

Variables	(1)	(2)	(3)	(4)	(5)	(6)	(7)	(8)	(9)	(10)	(11)	(12)
(1) PTS	1											
(2) ES	0.916^**^	1										
(3) AS	0.939^**^	0.772^**^	1									
(4) CS	0.941^**^	0.775^**^	0.862^**^	1								
(5) SE	0.708^**^	0.648^**^	0.632^**^	0.698^**^	1							
(6) BE	0.676^**^	0.625^**^	0.600^**^	0.666^**^	0.917^**^	1						
(7) EE	0.675^**^	0.614^**^	0.614^**^	0.661^**^	0.927^**^	0.778^**^	1					
(8) CE	0.602^**^	0.551^**^	0.531^**^	0.601^**^	0.917^**^	0.752^**^	0.781^**^	1				
(9) L2M	0.534^**^	0.502^**^	0.464^**^	0.525^**^	0.748^**^	0.652^**^	0.696^**^	0.719^**^	1			
(10) IS	0.436^**^	0.408^**^	0.386^**^	0.424^**^	0.623^**^	0.535^**^	0.573^**^	0.613^**^	0.858^**^	1		
(11) 0S	0.351^**^	0.336^**^	0.287^**^	0.356^**^	0.516^**^	0.455^**^	0.465^**^	0.505^**^	0.836^**^	0.554^**^	1	
(12) LE	0.580^**^	0.541^**^	0.516^**^	0.564^**^	0.772^**^	0.675^**^	0.741^**^	0.716^**^	0.843^**^	0.614^**^	0.549^**^	1

PTS, perceived teacher support; ES, Emotional support; AS, Autonomy support; CS, Competence support; SE, student engagement; BE, Behavioral engagement; EE, Emotional engagement; CE, Cognitive engagement; L2M, L2 Motivational self system; IS, ideal L2 self; OS, ought-to L2 self; LE, L2 learning experience. **p < 0.01.

### 3.4 Analysis of the mediating effects of L2 motivational self system

To address research question 3, this study constructed a structural equation model with the three types of L2 motivational self system as parallel mediating variables. From the fitting index of the statistical value, *X*^2^ = 5248.026, indicating a large chi-square value. And *X*^2^/df = 11.636 is larger than 3, which exceeds the range of model fit. Given the large sample size of this study and the fact that the chi-square value is very sensitive to the sample size, the results of the test utilizing the chi-square value and the *p*-value do not reflect well on the fitting effect of the structural equation model (Barrett, 2007). The present investigation involves a large sample (*N* = 2,633), which greatly affects the *X*^2^ and *X*^2^/df. Therefore, other multiple indicators should be taken into account for a comprehensive judgment. And the model had good fit indices for other fit indices, with CFI = 0.931 (>0.90); IFI = 0.931 (>0.90); TLI = 0.924 (>0.90); and RMSEA = 0.064 (<0.08), aligned with recommended thresholds (Marsh et al., 1988), which suggested a more favorable model fit. Moreover, the bootstrap method (5,000 iterations) was employed to assess the significance of mediating effects (Hayes et al., 2017) (Figure 1).

Table 4 revealed that perceived teacher support (*β* = 0.435, *p* < 0.001) was a direct positive predictor of student engagement, accepting H2. Perceived teacher support was a significantly positive predictor of ideal L2 self (*β* = 0.475, *p* < 0.001), L2 learning experience (*β* = 0.618, *p* < 0.001), and ought-to L2 self (*β* = 0.414, *p* < 0.001), accepting H1. Besides, ideal L2 self (*β* = 0.178, *p* < 0.001), L2 learning experience (*β* = 0.438, *p* < 0.001), and ought-to L2 self (*β* = 0.055, *p* < 0.001) were all positive predictors of student engagement, accepting H3.

**Table 4:** Results of path relationship test.

Pathway	Estimate	S.E.	C.R.	*P*
IS←PTS	0.475	0.030	23.036	***
OS←PTS	0.414	0.029	18.637	***
LE ← PTS	0.618	0.025	30.090	***
SE ← IS	0.178	0.012	23.235	***
SE ← OS	0.055	0.013	3.525	***
SE ← LE	0.438	0.018	21.720	***
SE ← PTS	0.435	0.012	23.235	***

PTS, perceived teacher support; SE, student engagement; L2M, L2 Motivational self system; IS, ideal L2 self; OS, ought-to L2 self; LE, L2 learning experience. ***p < 0.001.

**Figure 2:**
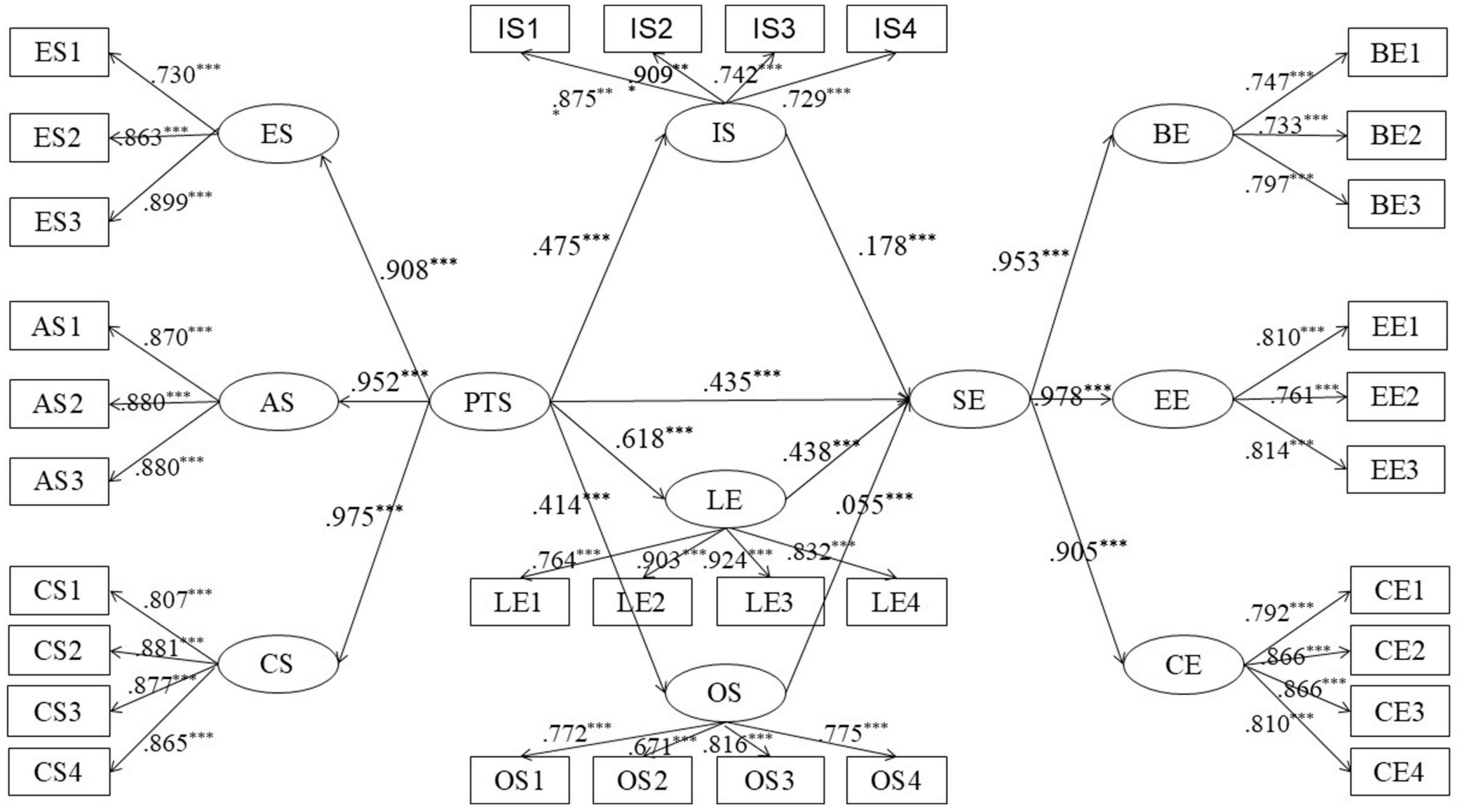
Structural model with standardized coefficients. PTS, perceived teacher support; ES, Emotional support; AS, Autonomy support; CS, Competence support; SE, student engagement; BE, Behavioral engagement; EE, Emotional engagement; CE, Cognitive engagement; IS, ideal L2 self; OS, ought-to L2 self; LE, L2 learning experience, ***p < 0.001.

As shown in Table 5 and Figure 2, perceived English teacher support directly predicted student engagement (*β* = 0.435, 95% CI [0.382, 0.486]), indicating that the direct path of “perceived English teacher support → student engagement” was reasonable. The results of the mediation effect analyses were as follows: the mediating effect of the L2 learning experience was significant (*β* = 0.271, 95% CI [0.235, 0.307]), suggesting that the indirect path of “perceived English teacher support → L2 learning experience → student engagement” was valid. The mediating effect of ideal L2 self was also significant (*β* = 0.084, 95% CI [0.064, 0.104]), indicating that the indirect path of “perceived English teacher support → ideal L2 self → student engagement” was valid; and the mediating effect of ought-to L2 self was also significant (*β* = 0.023, 95% CI [0.008, 0.038]), indicating that the indirect path “perceived English teacher support → ought-to L2 self → student engagement” was valid. Thus, H4 was accepted.

**Table 5:** Bootstrap analysis of the significance test of mediation effect.

Influence path	Estimate	S.E.	95% CIs		*p*	Relative effect (%)
			Lower	Upper		
Direct effect						
PTS → SE	0.435	0.026	0.382	0.486	***	53.506%
Indirect effects						
PTS → LE → SE	0.271	0.018	0.235	0.307	***	33.33%
PTS → IS → SE	0.084	0.010	0.064	0.104	***	10.37%
PTS → OS → SE	0.023	0.007	0.008	0.038	0.02*	2.83%

PTS, perceived teacher support; SE, student engagement; L2M, L2 Motivational self system; IS, ideal L2 self; OS, ought-to L2 self; LE, L2 learning experience. ***p < 0.001, **p < 0.01, *p < 0.05.

## 4 Discussion

### 4.1 Levels of perceived English teacher support, L2 motivational self system and student engagement of Chinese EFL undergraduates

The present study found that students generally perceived a high level of teacher support in English learning (Zheng et al., 2025). Among them, autonomy and competence support scores were high, consistent with Chi (2017), which suggests that teachers invested greater effort in supporting students to improve their autonomy and competence development. In addition, emotional support scored relatively low (Wang et al., 2025), suggesting that teachers did not place equal importance on the three types of support. Emotional communication with students can facilitate the establishment of a harmonious teacher-student relationship, which in turn has a positive impact on student engagement and achievement (Roorda et al., 2011), and therefore, teachers should provide more emotional support in teaching practice.

The participants generally had a medium-high level of the L2 motivational self system, indicating that most Chinese EFL undergraduates maintain a favorable attitude toward English learning (Lee and Lee, 2021; Thorsen et al., 2020). Ideal L2 self and L2 learning experiences are at a high level, which means that most students have clear goals and expect to satisfy their personal and professional development through learning English (Rattanaphumma, 2016), as well as experiencing a positive learning experience in the real learning process and gaining a certain sense of achievement and satisfaction (Ning and Downing, 2012). Ought-to L2 motivation was lower than the overall level, indicating that students are weakly driven by expectations from others or external pressures in English learning.

Besides, student engagement was generally at a high level, which aligns with previous studies (Liu et al., 2023), indicating that students generally have positive learning attitudes, effective learning strategies, and positive experiences in English classrooms. Emotional engagement is particularly prominent (Karaoğlan Yılmaz, 2022), indicating that students are able to experience positive emotional experiences and remain interested in English. However, behavioral engagement was relatively low, suggesting that Chinese EFL undergraduates were passive participants in English classrooms (Wang et al., 2025) and lacked the ability to actively engage in discussions or ask questions, which may limit their learning depth and student engagement. Therefore, teachers would develop effective teaching strategies to provide students with targeted support and guidance.

### 4.2 Relationships between perceived English teacher support, L2 motivational self system, and student engagement

This study explored the relationship and potential mechanisms between perceived teacher support and student engagement. The results showed students perceived more teacher support and more engagement in learning, which is consistent with the findings of another study (Liu et al., 2023). Further analysis of the dimensions of teacher support revealed that competence support was the strongest predictor of student engagement and was the predominant factor in the conceptualization of teacher support. Teachers can boost students’ learning effectiveness and engagement by communicating expectations, offering appropriate learning tools, and giving appropriate feedback (Chi, 2017). When students feel competent support, their motivation, academic achievement, and well-being increase, which in turn enhances student engagement. Therefore, it is advisable for teachers to focus on providing more competence support to effectively increase student engagement through specific teaching strategies. In addition, autonomy and emotional support were also strong predictors of student engagement. Autonomy support emphasizes teachers’ recognition of students’ subjectivity and promotes their autonomy in language learning and use. Compared with primary and high school students, undergraduates are more inclined to have more autonomy in their learning (Hensley et al., 2021). Tian and Wu (2019) found that Chinese students have limited opportunities to make choices in the classroom due to the teacher-driven teaching style. Therefore, teachers should be aware of students’ subjectivity and encourage them to learn independently, and they are more likely to be motivated to participate in foreign language learning when their autonomy needs are satisfied. Although the predictive effect of emotional support is relatively low, it still affects student engagement. Teachers are supposed to create a supportive and relaxing environment to promote teacher-student interaction and enhance students’ emotional and behavioral engagement (Guo et al., 2025).

As for the relationship between teacher support and the L2 motivational self systems, teacher support was positively correlated and a predictor of the L2 motivational self systems. L2 learning experience had the most significant effect size on student engagement. This is consistent with an existing study (Dornyei, 2019) indicating that teacher support can improve student engagement and boost their satisfaction with their learning experience. Teachers’ supportive behaviors, such as guidance, feedback, and encouragement in the English classroom, can satisfy students’ basic psychological needs of autonomy, competence, and relatedness, and thus stimulate students’ motivation (Xu X. et al., 2023). The effect of the ideal L2 self is more significant, which is consistent with the prior studies (Ghasemi, 2023). These findings reinforced that teacher support can effectively stimulate students’ intrinsic motivation and ideal goals, narrow the gap between current proficiency and future ideal self, and promote the students’ language learning to approach the ideal second language proficiency level gradually. Teacher support has a weaker correlation and a smaller effect size on ought-to L2 self. This may be interpreted by the possibility that the ought-to L2 self is more related to students’ external pressures, and social expectations (Dörnyei, 2005), which may be influenced by other factors such as family expectations, peer influences, or the Chinese educational environment (Li and Zhang, 2021; Ushioda and Dörnyei, 2017). The study found that incorporating teacher support into the L2 motivational self system theory further revealed the complex connection between teacher support and students’ L2 motivation in the context of EFL in China. This contributes to further refining and expanding the application of the L2 motivational self system in learning contexts, providing more pertinent recommendations for teaching.

The structural equation also confirmed that the L2 motivational self system played a significant function in predicting student engagement (Papi, 2010), revealing that the L2 motivational self system as a whole positively oriented student engagement. However, this study differs in its more comprehensive examination of the impact of three different types of L2 self-motivation on student engagement in China’s EFL context, particularly the ought-to L2 self. The L2 learning experience and ideal L2 self were positively connected to student engagement, which was consistent with the findings of Taguchi et al. (2009). This suggests that ideal L2 self and positive L2 learning experiences are key factors for motivation and can motivate students to be more engaged in foreign language learning. Additionally, the ought-to L2 self predicted student engagement, supporting the results of existing studies (Al-Hoorie, 2018; Li et al., 2025), which indicated that ought-to L2 self directly and positively predicted engagement. Manuel-Oronich et al. (2023) further pointed out that foreign language learning among Chinese non-English-major learners is driven by the ought-to L2 self. This may be associated with the “Chinese imperative,” which posits that Chinese students often develop extrinsic motivation to learn English to avoid academic failure and meet family expectations for a short time (You and Dörnyei, 2016). This ought-to L2 self is fundamentally tied to students’ sense of responsibility and obligation, which guides them to increased behavioral engagement in English learning. However, this current study reveals that while this motivational effect is statistically significant, its effect size remains weak, being depending on the degree of learners’ psychological internalization and the socio-cultural dynamics of the study environment (Dörnyei and Chan, 2013; Gong and Pang, 2025). Especially in China’s exam-oriented learning culture (Jiang and Dewaele, 2019; Xu J. et al., 2023), students perceive English learning as a burden to fulfill others’ expectations or to achieve exam success (Liu and Thompson, 2018), and they are prone to resistance, ultimately making it difficult to sustain engagement over extended periods. The study revealed the influence of various dimensions of L2 motivational self system on student engagement in the context of EFL in China, and further enriched the influencing factors of student engagement.

### 4.3 Multiple mediating effects of the L2 motivational self system

This study explored the mediating role of L2 motivation and emphasized the importance of English learning (Lee and Lee, 2021). It was found that the L2 motivational self system significantly mediated the effect between perceived English teacher support and student engagement. Perceived teacher support not only directly affects student engagement but also indirectly impacts it through the mediating function of the L2 motivational self system. This finding is consistent with prior research(Ghasemi, 2023), underscoring the pivotal function of teachers and their support in enhancing the learning experience and guiding students toward realizing their ideal L2 self, thereby promoting student engagement. According to the social cognitive theory (Bandura, 2001), student engagement is shaped by the interaction between internal factors (L2 motivational self system) and external environmental factors (teacher support), further confirming the significant mediator of the L2 motivational self system between teacher support and student engagement.

Further analysis revealed differences in the mediating strength of different types of L2 motivational self system. The indirect effects were mainly transmitted through L2 learning experiences (33.33%), indicating the important role of positive learning experiences in English learning (Huang, 2019), and with a small portion through ideal L2 self (10.37%) and ought-to L2 self (2.83%). In the structure of the L2 motivational self system, there is a powerful correlation between L2 learning experience, school context, and student engagement (Dörnyei, 2009; Ghasemi, 2023). Teachers, as important factors in the school context, can provide learners with competence support and emotional encouragement to enhance their positive learning experience (Sadoughi et al., 2023) and thus stimulate their motivation and engagement. The ideal L2 self is closely related to students’ interests and aspirations (Fathi and Hejazi, 2024) and can stimulate their intrinsic motivation (Safdari, 2021). However, in actual foreign language learning, students may find it difficult to transform their ideals into actual learning actions due to the lack of clear learning goals and improper learning methods. Teachers guide students to set ideal self goals and maximize their L2 self-motivation capacity to address the discrepancy between the actual self and the ideal self (Dörnyei and Ushioda, 2009). Ought-to L2 self is driven more by the expectations of others and external pressures, although this drive can promote student engagement, yet students may not develop their English level continuously because of external pressure or expectations rather than their inner love of learning English (Zhao et al., 2022). In short, this study emphasized the mediating function of L2 motivation by offering sufficient support, that is, teachers create positive learning contexts, guide students to set up goals, and transform their ought-toL2 selves into intrinsic motivation, to enhance their learning experiences and motivation, and facilitate student engagement finally.

## 5 Conclusion, implications, and limitations

In conclusion, the results emphasized the direct role of teacher support and the mediating role of the L2 motivational self system in improving student engagement, with L2 learning experiences exerting a greater mediating effect compared to the ideal L2 self and ought-to L2 self. Additionally, the present study further expands the scope of application of the L2 motivational self system theory in L2 learning contexts and provides empirical support for the application of the theory to different groups of learners and educational contexts. The findings highlight the essentiality of English teachers in stimulating students’ motivation and engagement in learning.

Pedagogically, these findings also present important practical implications for improving student engagement and English teaching quality. First of all, in foreign language teaching, teachers are required to provide sufficient emotional, autonomy, and competence support to promote student engagement. Specifically, teachers should give students positive attention, understand their needs, foster teacher-student interactions, and create a relaxing and enjoyable learning context to enhance students’ well-being and engagement (Klem and Connell, 2004). Furthermore, teachers are encouraged to actively transform their roles to provide abundant learning resources and targeted opportunities for choices to develop students’ self-regulated learning. Regarding competence support, teachers focus on offering tailored assistance based on students’ actual proficiency level and learning needs, and effectively improve student engagement through regular feedback and other instructional strategies (Chi, 2017).

Second, considering the pivotal role of motivation in English language learning (Muir, 2020), and the L2 motivational self system in particular, teachers can optimize the classroom context (Sadoughi and Hejazi, 2023) and design interactive teaching activities such as role-playing, group discussion, and extracurricular practical activities to provide students with opportunities for language practice while stimulating students’ interest and engagement. Moreover, teachers should also actively guide students to set up appropriate ideal L2 self goals, and stimulate students’ motivational regulation initiative and positivity through learning logs, English cultural activities, as well as, introducing successful role models (Fathi and Hejazi, 2024) to promote the sustainable development of second language learning. For the ought-to L2 self, teachers should pay attention to students’ external pressures and guide students to transform external expectations into students’ intrinsic learning motivation (Teimouri, 2017) through regular learning feedback, personalized learning suggestions, and encourage evaluations and other teaching strategies to enhance motivational sustainability.

Certainly, limitations were inevitable. Firstly, our study involved participants selected from several universities within a single province in southeastern China. The unbalanced distribution of participants in terms of gender may affect the results of this study to some extent. In subsequent empirical studies, the survey scope can be expanded to cover colleges from different regions and various types, while ensuring a more balanced gender distribution to better the credibility and wider applicability. Secondly, regarding the research methods, the self-survey report of the research participants may be affected by the complex interactions between the individuals and the sociocultural factors, potentially affecting the accuracy of the results. Future studies could incorporate qualitative research, such as classroom observation, reflective teaching logs, and interviews to enrich the observational perspectives and research methodology, to further analyze the relationship between these variables. Thirdly, this study only adopted a cross-sectional research design, which only examined the path of the influence of teacher support on student engagement at a specific point in time, and was unable to reveal the causal relationship among these variables, nor track the changes of the research participants over time. Future studies could adopt a longitudinal research design to conduct more in-depth dynamic investigations.

## Data Availability

The original contributions presented in the study are included in the article/supplementary material, further inquiries can be directed to the corresponding author/s.

## Footnotes

^1^ https://www.wjx.cn/
